# Prognostic Value of Malic Enzyme and ATP-Citrate Lyase in Non-Small Cell Lung Cancer of the Young and the Elderly

**DOI:** 10.1371/journal.pone.0126357

**Published:** 2015-05-11

**Authors:** Agnes Csanadi, Claudia Kayser, Marcel Donauer, Vera Gumpp, Konrad Aumann, Justyna Rawluk, Antje Prasse, Axel zur Hausen, Sebastian Wiesemann, Martin Werner, Gian Kayser

**Affiliations:** 1 Department of Pathology, Institute of Surgical Pathology, University Medical Center Freiburg, Breisacher Strasse 115a, D-79106 Freiburg, Germany; 2 Clinical Cancer Registry, Comprehensive Cancer Center Freiburg, University Medical Center Freiburg, Hugstetter Strasse 55, D-79106 Freiburg, Germany; 3 Department of Hematology and Oncology, University Medical Center Freiburg, Hugstetter Strassse 55, D-79106 Freiburg, Germany; 4 Department of Pneumonology, Hannover Medical School, Carl-Neuberg Strasse 1, D-30625 Hannover, Germany; 5 Department of Pathology, GROW-School for Oncology & Developmental Biology, Maastricht University Medical Center, Maastricht, The Netherlands; 6 Department of Thoracic Surgery, University Medical Center Freiburg, Hugstetter Strasse 55, D-79106 Freiburg, Germany; Queen Mary Hospital, HONG KONG

## Abstract

**Background:**

Lung cancer is the leading cause of death among malignancies worldwide. Understanding its biology is therefore of pivotal importance to improve patient’s prognosis. In contrast to non-neoplastic tissues, cancer cells utilize glucose mainly for production of basic cellular modules ‘(i.e. nucleotides, aminoacids, fatty acids). In cancer, Malic enzyme (ME) and ATP-citrate lyase (ACLY) are key enzymes linking aerobic glycolysis and fatty acid synthesis and may therefore be of biological and prognostic significance in non-small cell lung cancer (NSCLC).

**Material and Methods:**

ME and ACLY expression was analyzed in 258 NSCLC in correlation with clinico-pathological parameters including patient’s survival.

**Results:**

Though, overall expression of both enzymes correlated positively, ACLY was associated with local tumor stage, whereas ME correlated with occurrence of mediastinal lymph node metastases. Young patients overexpressing ACLY and/or ME had a significantly longer overall survival. This proved to be an independent prognostic factor. This contrasts older NSCLC patients, in whom overexpression of ACLY and/or ME appears to predict the opposite.

**Conclusion:**

In NSCLC, ME and ACLY show different enzyme expressions relating to local and mediastinal spread. Most important, we detected an inverse prognostic impact of ACLY and/or ME overexpression in young and elderly patients. It can therefore be expected, that treatment of NSCLC especially, if targeting metabolic pathways, requires different strategies in different age groups.

## Introduction

Lung cancer is the leading cause of malignancy related death worldwide [1]. Despite tremendous advances in medical therapies, prognosis of lung cancer patients remains poor with a 5-year survival rate ranging between 7.9% and 16.5% [1, 2].

By showing that patients benefit from different chemotherapy regimens dependent on histological subtype, Scagliottti abolished the dogma to treat non-small cell lung cancer (NSCLC) as an oncologically homogenous group [3]. Thus, the major NSCLC subgroups, adenocarcinoma (LAC), squamous cell carcinoma (SCC) and large cell carcinoma (LCC) do not only show different histological patterns, but present specific biological and molecular features, too. A better insight into these distinct characteristics will aid to direct personalized therapies. In this context, metabolic changes associated with malignant cellular transformation are of pivotal importance.

For decades, it is known that malignant tumors produce excessive lactate even in the presence of sufficient oxygen (Warburg effect) [4]. Yet, mechanisms behind this phenomenon are not fully understood. Malignant cells function with metabolic autonomy, and glucose, its metabolites as well as glutamine are not only energy sources. They also serve as basic building units via generation of key molecules needed for cellular and thus malignant tumor growth [5, 6].

As enzymes of glucose metabolism represent a common downstream endpoint for various tumor driver mutations, they could be promising targets for new chemotherapeutic agents, too [7]. Among these enzymes, ATP-citrate lyase (ACLY) and malic enzyme (ME) are two key players: ME serves as source of reductive equivalents in highly cataplerotic malignant cells. ACLY builds a physiological shunt between glucose metabolism and fatty acid synthesis [6].

We therefore analyzed the expression patterns of these two enzymes to elucidate their association with clinico-pathological features and their biological impact on patient’s survival in NSCLC. Our results clearly show that functional metabolic changes in NSCLC are complex, differ in histological subtypes and predict different outcomes depending on patient’s age.

## Materials and Methods

### Ethics statement

The study has been approved by the University Medical Center Freiburg (Ethics committee University Medical Center Freiburg, EK 10/12). Patient related data has been pseudonymized and results obtained by this study did not influence patient’s treatment. Archived material had been used at least three years after initial diagnosis. By signing the treatment contract with the University Medical Center Freiburg, each patient agrees that his/her pseudonymized tissue(s) may be suspect to retrospective research trials not interfering with or influencing current treatment options. The ethics committee of the University Medical Center Freiburg thus approved that no individual study specific consent of each patient had to be obtained.

### Cohort

258 patients suffering from NSCLC were included in this study. Patients underwent surgical treatment between 1990 and 2007 (Department of Thoracic Surgery, University Medical Center Freiburg; S1 Dataset) and had not received neoadjuvant therapy. Fixation, grossing and paraffin embedding were performed according to routine protocols. All cancer cases were reclassified according to the current WHO classification [8], staging was reassessed in concordance with the latest UICC classification [9]. Tissue multi arrays (TMA) were constructed with a core diameter of 2 mm. From all specimens three TMA-cores were taken from different sites to avoid bias from intratumoral heterogeneity. A TMA of 36 corresponding non-neoplastic lung tissues served as control set. (S1 Table: summary of clinic-pathological data).

### Immunohistochemistry and scoring

Heat-induced antigen retrieval was performed at pH 9.0 for ACLY and at pH 6.0 for ME. Primary antibody incubation time was 30 minutes (ACLY: 1:400, Cell Signaling Technologies 4331S; ME: 1:2000 dilution, Clone 3H5, Abnova Biozol). Visualization was performed by alkaline phosphatase with Fast Red-type chromogen (DAKO REAL K5005) and horseradish peroxidase with diaminobenzidine based chromogen (DAKO FLEX EnVision) for ME and ACLY, respectively. Nuclear counterstaining was conducted with hematoxilin (Mayer’s acidic haemalaun, Waldeck, catalog no. 1A-552). The DAKO autostainer platform was used for staining procedures.

For both immunohistochemical stains, ACLY and ME, protocols were validated for specific staining by omission of the primary antibodies. These validation procedures did not show unspecific chromogen reactions.

**Fig 1:**
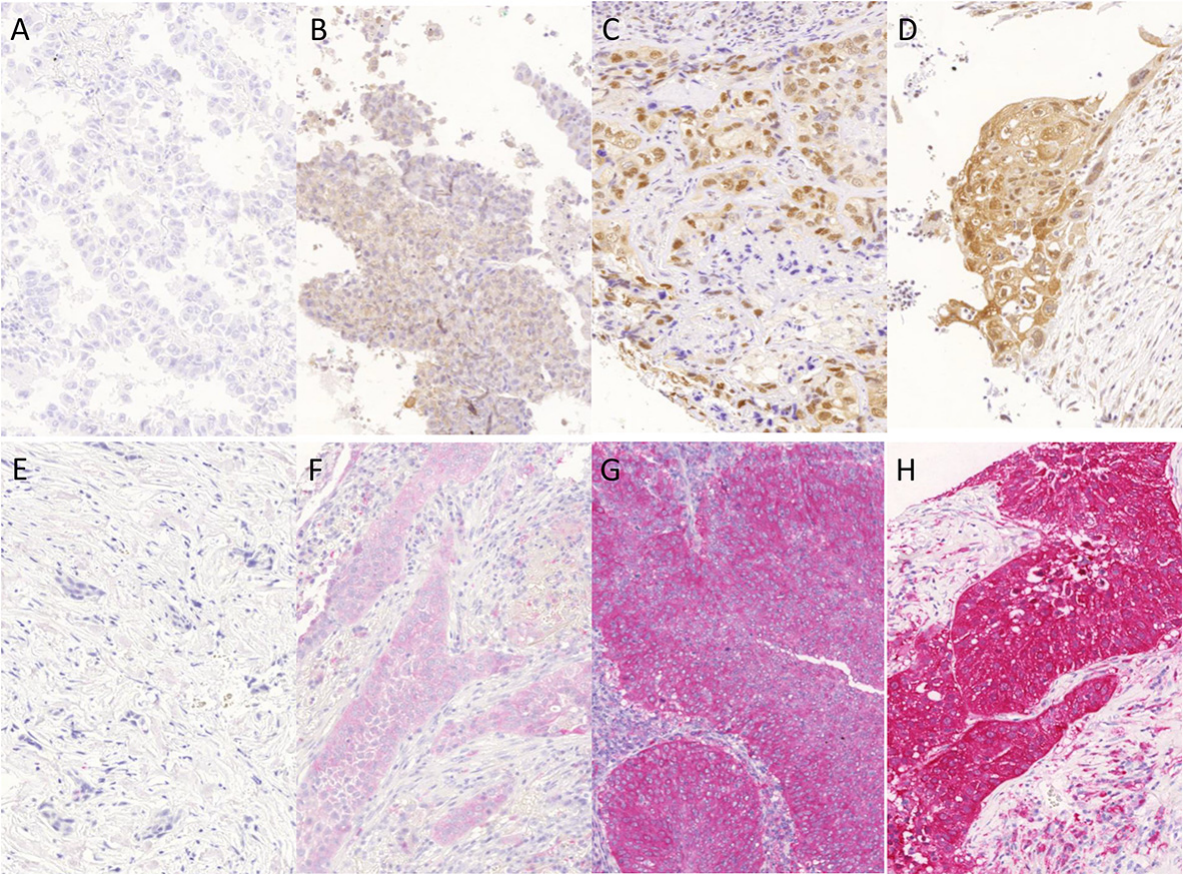
Immunohistological expression of ACLY (A – D) and ME (E – H). (A)–(D) Specific ACLY-expression was detectable in the nucleus, or in the cytoplasm. (A); tumor tissue without specific ACLY staining—intensity score = 0; (B) weak specific cytoplasmic ACLY staining—intensity score = 1; (C) moderate nuclear ACLY staining—intensity score = 2 and moderate cytoplasmic ACLY staining—intensity score = 2; (D) strong cytoplasmic ACLY staining—intensity score = 3, additionally moderate nuclear staining is present; (E)–(H) Specific cytoplasmic expression of ME, (E) tumor tissue without specific cytoplasmic ME staining—intensity score = 0 (F); weak specific cytoplasmic ME staining—intensity score = 1 (G); moderate cytoplasmic ME staining—intensity score = 2 (H) and with strong specific cytoplasmic ME staining—intensity score = 3 (E). (Magnification; 20x)

Enzyme expression was considered positive, if specific cytoplasmic staining was detected. For ACLY, specific nuclear positivity was also assessed. Immunohistochemical scoring followed previously described protocols [10, 11] and was evaluated in analogy to internationally accepted scoring of predictive markers [12, 13]. Staining intensity was evaluated semi-quantitatively using a 4-tired scoring system (Fig 1). Percentage of positive tumor cells was determined by considering all positive tumor cells in relation to their absolute number. Percentage figures were rounded to the next decimal. Nuclear and cytoplasmic expressions of ACLY were evaluated separately.

### Statistics

For all statistical analyses mean values of the three TMA-cores of each case were used. Differences in enzyme expression were evaluated by non-parametric tests.

Survival analysis included Kaplan-Meier curves and log-rank tests. For multivariate analyses, Cox-regression models were used. All statistical analyses were performed using the SPSS 21.0 software suite. Level of significance was set to 5% (i.e. *p*<0.05). The overall level of significance has been adjusted for multiple testing using the Benjamini-Hochberg method [14] (S2 Table).

## Results

### ACLY and ME are upregulated in NSCLC

ACLY expression in tumor tissue was detected in both, cytoplasm and nucleus (Fig 1), whereas ME was detectable only in the cytoplasm (Fig 1). Immunohistochemical enzyme expression was significantly higher in tumor cells (ACLY nucl.: 19.92 +/- 21.57; ACLY cytopl.: 22.23 +/- 21.57; ME: 52.49 +/- 37.86) than in corresponding non-neoplastic lung tissue (ACLY nucl.: 16.57 +/-16.75; ACLY cytpl.: 11.43 +/- 20.97; ME: 9.53 +/- 10.50; *p* < 0.001)

### ME but not ACLY is differentially expressed in histological NSCLC subtypes

As differentiation between LAC and SCC of the lung is of therapeutic importance, we analyzed expression patterns in relation to these two histological NSCLC subtypes. ME expression was higher in SCC compared to LAC (*p* < 0.001). ACLY did not show a significant correlation with histological subtype. Furthermore, a significantly higher expression only of ME was found in smokers compared to non-smokers (*p* = 0.012).

### ACLY but not ME expression is associated with local tumor stage

Analyzing immunohistological patterns of ACLY and ME in correlation with local tumor stages, i.e. pT, we observed that ACLY expression was significantly lower in advanced pT-stages. Though a significant positive overall correlation of ME and ACLY was found (nuclear ACLY—ME: correlation coefficient = 0.179, *p* < 0.001; cytoplasmic ACLY—ME: correlation coefficient = 0.155, *p* = 0.001), ME expression did not show a correlation with local tumor stages. Analog results were observed for ACLY expression in correlation with metric tumor size (ACLY cytoplasmic: correlation coefficient -0.135, *p* = 0.003; ACLY nuclear: correlation coefficient: -0.144, *p* = 0.001; ME: correlation coefficient = 0.006, *p* = 0.887). While infiltration of the visceral pleura also contributes to local tumor stage, no statistically significant correlation was observed regarding pleural invasion (ACLY cytoplasmic: *p* = 0.186; ACLY nuclear: *p* = 0.532; ME: *p* = 0.971).

### ME but not ACLY expression is associated with mediastinal metastatic events

To investigate the relationship of ME and ACLY expression with systemic tumor expansion, we separately analyzed NSCLC of nodal positive patients in correlation with location of lymph node metastases. The presence of mediastinal lymph node metastases was significantly correlated with higher expression of ME in primary tumor tissue (*p* = 0.041) but not of ACLY (cytoplasmic: *p* = 0.511; nuclear: *p* = 0.446). This association was particularly strong in LAC (ME: *p* = 0.030).

### Overexpression of either ME, ACLY or both is an independent prognostic factor

**Fig 2:**
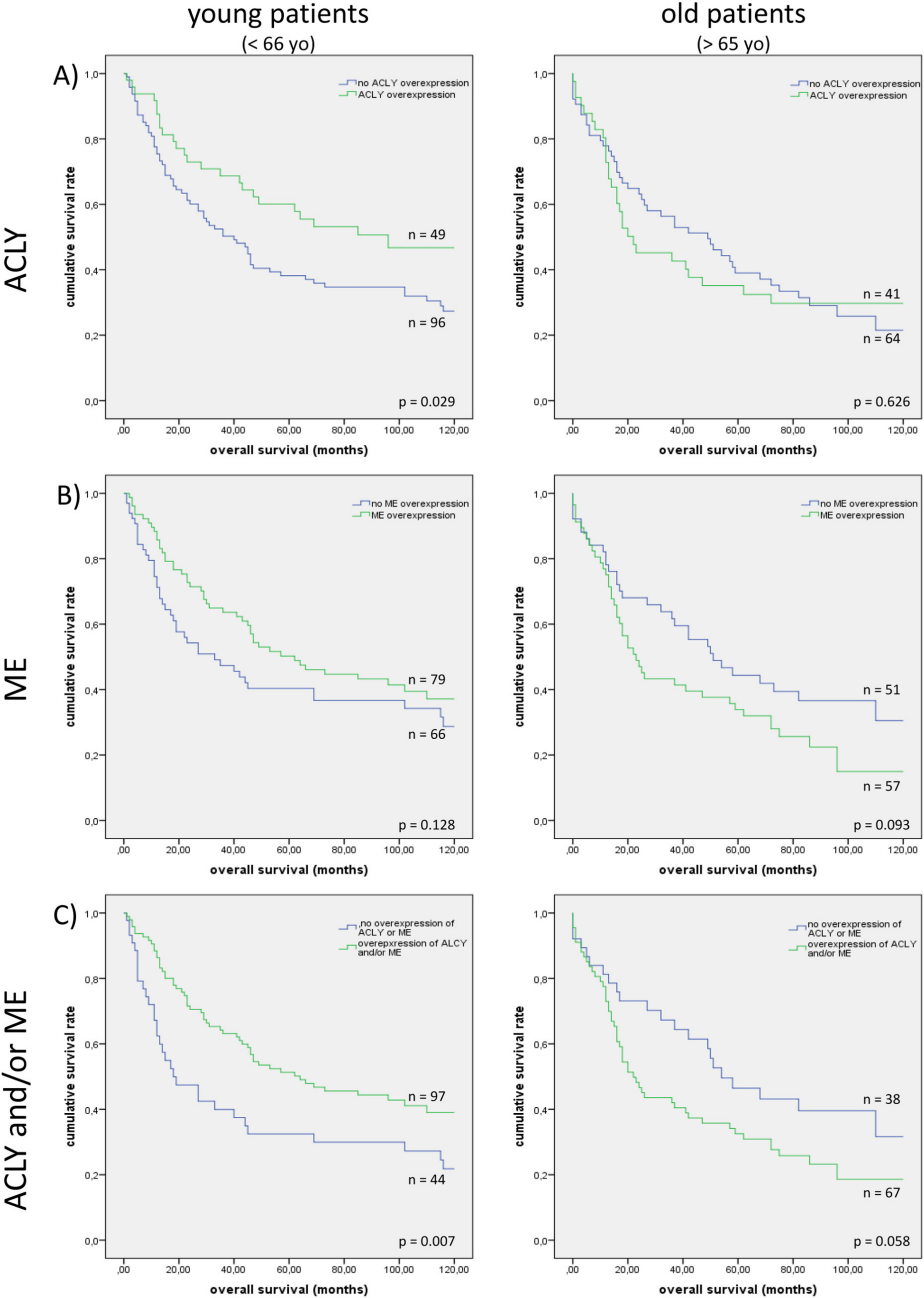
Overexpression of ACLY and ME in correlation with patient’s overall survival. A) Overexpression of ACLY is associated with a better outcome in young patients but not in older patients. B) ME overexpression shows a statistical trend towards a poorer overall survival in older patients. C) Young patients whose tumors revealed either ACLY and/or ME overexpression had a significantly longer overall survival compared to those without overexpression of either enzyme. But in older patients overexpression of either ACLY and/or ME may be associated with poorer overall survival.

To avoid statistical bias, mean values of ME and ACLY expression were used for dichotomization. In the overall analysis no significant correlation of ME and ACLY overexpression with patient’s survival was found. Similar results were obtained in subgroup analyses according to smoking habits, sex, histological grading, pT- or pN-stages. Age stratification was performed by using the median age (65 years) at NSCLC diagnosis. No significant correlation between age and pT, pN or overall UICC stage was detected. In young patients, nuclear ACLY overexpression proved to be associated with a favorable overall survival (*p* = 0.029), while this was not the case in patients older than 65 years (*p* = 0.626). ME overexpression in these two age subgroups only showed a statistical trend in older patients (*p* = 0.093). Young patients with overexpression of ME or nuclear ACLY or both in their tumors, had a significantly longer overall survival compared to those without overexpression of these enzymes (*p* = 0.007; Fig 2). Multivariate analysis, which included UICC stage, the only additional prognosticator in this patient group, proved this to be an independent prognostic factor (*p* = 0.002; Table 1). On the other hand, overexpression of either or both of the two enzymes resulted in shorter overall survival in older patients (Fig 2, *p* = 0.058).

**Table 1:** Summary of survival analysis of ACLY- and ME overexpression stratified by age.

		Hazard ratio	95% CI	Univariate log-rank test	Multivariate Cox regression
**Young (< 66 yo)**	ACLY	0.599	0.375–0.957	p = 0.029	p = 0.230
	ME	0.723	0.474–1.103	p = 0.128	p = 0.018
	ACLY and/or ME	0.556	0.359–0.861	p = 0.009	p = 0.002
**Older (> 65 yo)**	ACLY	1.124	0.988–1.809	p = 0.626	p = 0.386
	ME	1.486	0.930–2.373	p = 0.093	p = 0.140
	ACLY and/or ME	1.616	0.975–2.677	p = 0.058	p = 0.072

Multivariate cox regression analysis reveals ACLY- and/or ME overexpression as an independent prognostic factor in young NSCLC patients (< 66 years of age).

## Discussion

Due to its high incidence and mortality, lung cancer still remains one of the major health burdens, worldwide. It is therefore of pivotal importance to better understand its biology in order to develop new suitable treatment options.

The metabolic switch of tumor cells to aerobic glycolysis is a well-known event. On the one hand, it serves to facilitate the uptake and incorporation of nutrients into basic cellular building blocks. On the other hand, it results in the production of lactate, which facilitates metastasis formation and therapy resistance [15, 16].

In several malignant tumors it has been shown that ACLY is not only elementary for de-novo fatty acid synthesis [17], but also its rate limiting step [18–21]. As one of the key enzymes of de-novo fatty acid synthesis, ACLY generates cytosolic acetyl-coenzyme A (acetyl-CoA) [22, 23] and oxaloacetate. The latter is reduced to malate by malate dehydrogenase. The cytosolic isoform of malic enzyme converts malate into pyruvate [24]. Pyruvate that is not shuttled into the mitochondrion to generate oxaloacetate, is further converted into lactate [6]. ACLY and ME expression may therefore be altered in malignant tumor cells compared to non-neoplastic tissues. Comparing non-neoplastic lung and NSCLC tissue, we found a statistically significant increase of both, ACLY and ME expression within neoplastic cells. This is in concordance with other findings concerning altered carbohydrate metabolism in cancer [10, 11, 25–27]. In our cohort, ME and ACLY expression, nuclear as well as cytoplasmic, showed a positive correlation. The fact that correlation coefficients are rather small may reflect complex interrelations between several metabolic enzymes as well as high histomorphological heterogeneity of NSCLC. Comparison of immunohistochemical expression patterns of ME and ACLY further supports this statement as only ME-expression significantly differed between LAC and SCC. This kind of differential expression patterns in NSCLC subtypes has also been shown for other enzymes related to altered cellular cancer metabolism [10, 11, 28].

Since SCC is often found in heavy smokers and LAC are so called typical non-smoker carcinomas, significant higher expression of ME in tumors of patients with smoking history is not surprising. This can be due to different hypoxic states of tumors and/or patients leading to different metabolic states in NSCLC and most probably in SCC compared to LAC, too. The majority of smoking associated NSCLC possess p53 mutations and thus, the frequency of p53 mutations in SCC is higher compared to LAC [29]. Recently, Jiang could verify that ME expression is regulated by p53. According to their findings, p53 is responsible for down regulating ME expression [30]. These findings are in good concordance with ours, that ME is not only overexpressed in NSCLC compared to tumor free lung tissue but also, that ME expression is higher in SCC compared to LAC and in smokers compared to non-smokers.

Striking to us was, that ACLY and ME revealed different expression patterns dependent on local or mediastinal tumor spread. While ACLY was negatively correlated with local tumor extension measured by pT-stage, as well as metric tumor size, ME only showed a significant correlation with mediastinal metastatic events in comparison to hilar lymph node metastases (pN1 vs pN2/pN3). Changes in tumor metabolism, therefore, seem to be complex and may be different not only in histological subtypes but also according to local or systemic tumor spread.

Furthermore, in recent research results, additional functions of ACLY beside involvement in glucose metabolism have been detected: ACLY is also a key-player in histone acetylation. These findings suggest a link between growth factor changes in cancer metabolism and gene expression which is realized by ACLY [31]. In analogy to Wellen, Londono Gentile just recently published that ACLY at least in part regulates DNA methyltransferase-1 (DNMT1) [32]. Different localization of ACLY may therefore reflect different activities within the cell, i.e. cytoplasmic ACLY is predominantly involved in cancer cell metabolism, while nuclear ACLY is predominantly involved in regulation of gene expression [31, 33]. For this reason, we assessed different localizations of ACLY, i.e. nuclear and cytoplasmic, separately. In concordance with our results, Migita showed that ACLY was significantly higher expressed in LAC compared to non-neoplastic lung tissue [22]. In their publication, ACLY was also a prognostic factor, while they showed that high levels of ACLY were associated with a poorer outcome [22]. We could not reproduce this finding of Migita. But in contrast to their results, nuclear ACLY overexpression was of significant benefit in young patients of our cohort. Compared to Migita, we included not only LAC but also SCC as well as LCC and immunohistochemical analysis of ACLY expression was assessed not only for staining intensity but also according to the fraction of positive cells and with regard to subcellular localization of ACLY. In addition to these analytical differences, Migita investigated the expression of phosphorylated ACLY, whereas our antibody is directed against ACLY regardless its phosphorylation status.

Furthermore, in our detailed subgroup analyses, we could show that patient’s age may also influence NSCLC biology. While high levels of either ACLY and/or ME were a good prognostic factor in patients younger than 65 years, a statistical trend to the opposite was detected in elderly patients. This may indicate different changes in tumor metabolism and enzyme function. Several metabolic changes are implicated with older age, such as increasing hypoxia and higher incidence of diabetes mellitus type 2. In a large cohort study, type 2 diabetes mellitus was associated with a higher risk to develop several types of cancer, including lung carcinoma [34]. In this context, both, ACLY and ME have been shown to be important for glucose related insulin secretion [24]. By the fact, that incidence of type 2 diabetes as well as generalized hypoxia due to lung function impairment is higher in older patients, our findings of changing impact of ACLY and ME enzymatic fitting in NSCLC can be functionally supported. Diversity of NSCLC in different age populations is known for genetic aberrations and incidences of driver mutations [35, 36]. Thus, our findings, that changes of metabolic enzymes can be of different influence on cancer biology in NSCLC, further support that lung cancer arising in young patients may be biologically and genetically different from those of older patients.

In vitro and in vivo studies indicate that new pharmacological agents inhibiting ACLY can lead to significant decrease in cellular and tumor growth [22, 33, 37, 38]. Since our results show that overexpression of ACLY and/or ME in patients older than 65 years of age tend to have a poorer prognosis, they suppose that older patients may profit most from these ACLY inhibitors.

Concluding, expression patterns of the metabolic enzymes ACLY and ME are of different biological impact on survival in NSCLC patients. While in young patients overexpression of either ACLY or ME is indicative for a favorable overall survival, it tends to have the opposite effect in older patients. With the development of new inhibitory drugs directed against ACLY, our results support new treatment options with special focus on aged NSCLC patients.

## Supporting Information

S1 Table Summary of clinico-pathological data. (DOCX)

S2 Table Summary of the presented relevant statistical test results adjusted for multiple testing. (DOCX)

S1 Dataset Complete dataset. (XLS)
