# Supplementary material for: Prognostic Value of Malic Enzyme and ATP-Citrate Lyase in Non-Small Cell Lung Cancer of the Young and the Elderly
Source: PLoS One. 2015 May 11;10(5):e0126357. doi: 10.1371/journal.pone.0126357 (PMC4427316; doi:10.1371/journal.pone.0126357)
Supplement: S1 Table — (DOCX) [file pone.0126357.s001.docx]

**Supplementary table S1**: Summary of clinico-pathological data

|  | **Median** | **Range** |
| --- | --- | --- |
| Age at diagnosis | 65 years | 35 - 83 |
| Overall survival after diagnosis | 37 months | 0 - 120 |
|  |  |  |
|  | **Number** | **Percent** |
| **Sex** |  |  |
| Male | 183 | 71 % |
| Female | 75 | 29 % |
|  |  |  |
| **Smoking habits** |  |  |
| Non-smoker | 31 | 12 % |
| smoker | 184 | 71 % |
| Not assessable | 43 | 17 % |
|  |  |  |
| **Histological tumor type** |  |  |
| LAC | 102 | 39 % |
| SCC | 95 | 37 % |
| LCC | 61 | 24 % |
|  |  |  |
| **pT** |  |  |
| pT1a | 31 | 12 % |
| pT1b | 35 | 14 % |
| pT2a | 88 | 34 % |
| pT2b | 34 | 13 % |
| pT3 | 46 | 18 % |
| pT4 | 24 | 9 % |
|  |  |  |
| **pN** |  |  |
| pN0 | 142 | 55 % |
| pN1 | 37 | 14 % |
| pN2 | 71 | 28 % |
| pN3 | 2 | 1 % |
| Not assessable | 6 | 2 % |
|  |  |  |
| **Disease stage (UICC)** |  |  |
| IA | 42 | 16 % |
| IB | 49 | 19 % |
| IIA | 39 | 15 % |
| IIB | 27 | 11 % |
| IIIA | 70 | 27 % |
| IIIB | 13 | 5 % |
| IV | 13 | 5 % |
| Not assessable | 5 | 2 % |
