# Supplementary material for: Prognostic Value of Malic Enzyme and ATP-Citrate Lyase in Non-Small Cell Lung Cancer of the Young and the Elderly
Source: PLoS One. 2015 May 11;10(5):e0126357. doi: 10.1371/journal.pone.0126357 (PMC4427316; doi:10.1371/journal.pone.0126357)
Supplement: S2 Table — (DOCX) [file pone.0126357.s002.docx]

**Supplementary Table S2.** Summary of the presented relevant statistical test results adjusted for multiple testing.

| **Item tested** | **p-value** | **Local p-value** [14] |
| --- | --- | --- |
| NSCLC vs non-neoplastic lung tissue | < 0.001* | 0,00192 |
| Correlation of nuclear ACLY and ME expression | < 0.001* | 0,00400 |
| Correlation of ME expression and histological tumor type | < 0.001* | 0,00625 |
| Correlation of cytoplasmic ACLY and ME expression | 0,001* | 0,00870 |
| Correlation of nuclear ACLY expression and metric tumor size | 0,001* | 0,01136 |
| Multivariate Cox regression analysis of ACLY-expression in patients < 66 years | 0,002* | 0,01429 |
| Correlation of cytoplasmic ACLY expression and metric tumor size | 0,003* | 0,01750 |
| Log rank survival analysis of ACLY and/or ME overexpression in patients < 66 years | 0,007* | 0,02105 |
| Correlation of ME expression and smoking history | 0,012* | 0,02500 |
| Log rank survival analysis of nuclear ACLY overexpression in patients < 66 years | 0,029* | 0,02941 |
| Correlation of ME expression and presence of lymph node metastases in LAC | 0,030* | 0,03438 |
| Correlation of ME expression and presence of lymph node metastases | 0,041 | 0,04000 |
| Log rank survival analysis of ACLY and/or ME overexpression in patients > 65 years | 0,058 | 0,04643 |
| Log rank survival analysis of ME overexpression in patients > 65 years | 0,093 | 0,05385 |

* statistically significant after adjustment for multiple testing [14]
